# Strategies to improve the quality of reporting nursing research

**DOI:** 10.4069/kjwhn.2022.06.08.1

**Published:** 2022-06-21

**Authors:** Ju-Eun Song

**Affiliations:** College of Nursing, Ajou University, Suwon, Korea

Research that demonstrates high quality reporting is important to improve clinical nursing practice and facilitate follow-up studies in various disciplines. Poor reporting is unethical [1,2], and such papers are often confusing, resulting in impractical, futile information that can even be detrimental to patient care [1,3]. Therefore, quality journals are invested in publishing good papers with good reporting quality, often maintained effectively via a peer review system [3,4]. However, the effective use of reporting guidelines is also considered a useful strategy to enhance the reporting quality of research published in academic journals [3,5].

The most frequently recommended and well-known reporting guidelines were developed by the EQUATOR Network (https://www.equator-network.org/). EQUATOR is an acronym for Enhancing the QUAlity and Transparency Of health Research. As the name suggests, the EQUATOR Network, an international organization, is a new initiative to persuade clear and transparent health research reporting [2,6]. It aims to ensure accuracy, completion, and transparency in reporting health research studies to promote functionality and replicability of research and to make health research credible and valuable by popularizing reporting guidelines for health research [6,7].

The *Korean Journal of Women Health Nursing* (KJWHN), as the official journal of the Korean Society of Women Health Nursing, is constantly striving to improve the journal quality by publishing quality research reports. Not only is this beneficial for our readership, but this would also be one of the strategies for the journal to be indexed in international journal databases, such as the Social Science Citation Index (SSCI). As one of the efforts to improve the quality of reporting of published research, while adopting a double-blind peer review system comprising a professional reviewer pool, KJWHN also recommends using the reporting guidelines of the EQUATOR Network in its author guidelines [8]. In addition, the editorial board of KJWHN has published three articles analyzing adherence to reporting guidelines of research published in KJWHN and highlighted areas for reporting improvement [9-11]. KJWHN’s effort to adopt and inform the reporting guidelines of the EQUATOR Network is advanced level compared to other journals in Korea.

Despite the concerns regarding the strict use of reporting guidelines inhibiting the creativity of researchers [12], the perceived value of reporting guidelines has increased, and adopting reporting guidelines has become an international trend and principle in manuscript writing. Nevertheless, difficulties in correctly selecting and using guidelines according to research design have been reported [13,14]. Therefore, in this editorial, to improve the use of reporting guidelines, I want to (1) introduce the EQUATOR Network and reporting guideline development; (2) analyze the status of recommendations for reporting guidelines in nursing journals; (3) explain how to find appropriate reporting guidelines considering the research design; and (4) suggest strategies to efficiently use reporting guidelines.

## The EQUATOR Network and reporting guidelines development

The EQUATOR Network was officially launched in 2008 as a result of the EQUATOR project, which was funded by the United Kingdom in 2006 and intended to make a map for preparing and disseminating health research reporting guidelines and establish a global collaborative relationship among key persons [6]. Twenty-seven key individuals including representatives of reporting guideline development groups, journal editors, peer reviewers, and funders from 10 countries collaborated on the project. The EQUATOR Network also established four national centers between 2014 and 2016, to focus on activities that raise awareness and support good research reporting practices [6].

As a simple structured tool, reporting guidelines are used by researchers while writing manuscripts and they comprise a basic list of information required to enable reviewers and readers to make accurate appraisal of the research quality [6]. As such, they are tools that help achieve good quality reporting in health studies. Adhering to reporting guidelines properly can also facilitate accurate replicating by other researchers and effective use by nurses and healthcare professionals to make a better clinical decision [4-6]. Currently, there are hundreds of general guidelines according to research types and special guidelines, which are expanded forms of general reporting guidelines.

## The status of recommendations for reporting guidelines in nursing journals

In addition to many biomedical journals, nursing journals also emphasize the use of reporting guidelines while preparing manuscripts, but there are no previous studies on how much the reporting guidelines are specifically recommended in nursing journal guidelines. Therefore, for this editorial, 122 journals listed in the nursing category of SSCI as of June 1, 2022 (Supplementary material 1), were analyzed in terms of reference to the reporting guidelines of the EQUATOR Network.

Among the 122 SSCI nursing journals, 94 journals (77.0%) specified adopting the EQUATOR Network’s guidelines in their author guidelines, whereas 28 journals (23.0%) did not mention reporting guidelines at all. This rate is quite high compared to a 2012 study of journals in other disciplines, in which 46% of journals mentioned reporting guidelines in their journal instruction [3]. However, a sizable proportion of nursing journals still require improvement.

Among the 94 journals that mentioned reporting guidelines use, 85 (90.4%) provided information about representative reporting guidelines according to the research design, such as CONSORT (CONsolidated Standards Of Reporting Trials) guidelines along with the EQUATOR Network link URL, whereas 9 journals (9.6%) simply mentioned the use of the reporting guidelines of the EQUATOR Network without listing reporting guidelines specifically.

The reporting guidelines that were frequently mentioned more than 10 times in nursing journals are PRISMA (Preferred Reporting Items for Systematic reviews and Meta-Analyses) for systematic review (n=71), CONSORT for randomized trials (n=64), SQUIRE (Standards for QUality Improvement Reporting Excellence) for quality improvement studies (n=42), STROBE (STrengthening the Reporting of OBservational studies in Epidemiology) for observational studies (n=41), COREQ (COnsolidated criteria for REporting Qualitative research) for qualitative research (n=37), TREND (Transparent Reporting of Evaluations with Nonrandomized Designs) for nonrandomized trials (n=27), STARD (STAndards for the Reporting of Diagnostic accuracy) for diagnostic/prognostic studies (n=19), CARE (CAse REport) for case report (n=14), SRQR (Standards for Reporting Qualitative Research) for qualitative research (n=14), and MOOSE (Meta-analysis Of Observational Studies in Epidemiology) for systematic review of observational studies (n=12), as arranged based on their frequency. Details, including the meaning of acronyms for reporting guidelines, version information, and the direct link URL of either the EQUATOR website or individual guideline website; are presented in Table 1.

If readers click the suggested link URL in Table 1, they can easily find information about an updated or previous version of the reporting guideline, related forms (e.g., checklists or flow diagrams), history of guideline development, and related or elaborated publications to state the development process and provide a detailed explanation for correct use. In addition, reporting guidelines can be downloaded in either PDF (portable document format) or word file. Although following a reporting guideline does not guarantee acceptance for publication, it is the initial step for the successful publication of a manuscript [15].

In addition, the level of recommendation regarding reporting guidelines also varied. Some nursing journals required authors to complete and attach reporting guideline checklists while submitting the manuscript, whereas other journals only encouraged authors or reviewers to refer to reporting guidelines when writing or reviewing a manuscript, and as stated above, some journals did not mention reporting guidelines at all. Given that this is a phenomenon also frequently seen in other disciplines [4], although reporting guideline use in nursing journals is not low compared to that in other disciplines, the recommendations for using reporting guidelines in author instructions should be extended further.

## How to find reporting guidelines to fit the research design

Numerous guidelines have been presented on the homepage of the EQUATOR Network [6], but it is not easy to find guidelines suitable for the study design. It has been reported that many authors struggle to follow reporting guidelines, especially in choosing the right guidelines for their study and in using them correctly [7], because while many journals mention reporting guidelines as general statements rather than suggesting clear instructions about how to select or use them [8]. Thus, this is one of the main challenges in improving the use of reporting guidelines, which EQUATOR is trying to solve. To promote the correct use of reporting guidelines, improving the author’s understanding of report guidelines according to the research design and increasing the motivation for using those guidelines are important.

Recently, algorithms and websites have been developed to help authors find reporting guidelines that fit their research design effectively. The EQUATOR Network developed the EQUATOR Reporting Guidelines Decision Tree (https://www.equator-network.org/wp-content/uploads/2013/11/20160301-RG-Decision-Tree-used-for-EQUATOR-wizard-vn-1.pdf), which is an algorithm that helps in the selection of reporting guidelines [12], and the EQUATOR Wizard (https://www.penelope.ai/equator-wizard), a new tool to help authors find the right reporting guideline or different checklists for different types of study design [13]. The UK EQUATOR Center also launched GoodReports.org, a website that aids authors in finding and using reporting guidelines [16]. Various electronic algorithms are currently being developed to facilitate the choice of correct reporting guideline(s), and other tools are being integrated into journal editorial management processes [1,2]. I suggest that all readers, including authors and reviewers, visit the suggested sites to determine the appropriate guidelines for their research design.

## Strategies for better use of reporting guidelines for all users

As the positive influence of adhering to reporting guidelines enhancing the quality of published research is evident, I suggest strategies for the effective use of reporting guidelines by authors, reviewers, and journal editors, based on the EQUATOR Network’s suggestions and previous studies.

Firstly, for authors, try to find out the reporting guidelines when planning the study and drafting the manuscript, rather than at the stage of submitting the manuscript [16]. Authors should be encouraged to also check any new relevant guidelines that are more compatible with their research topic because there are many extended versions of the general guidelines according to the study design, which continue to be finessed and developed. In addition, authors should ensure adherence of all items in the reporting guidelines before submitting their manuscript and if not, explain why some items were not reported in their manuscript. The authors should note that research must always be reproducible.

Secondly, reviewers should understand the reporting guidelines correctly and verify the proper reporting of each item in the manuscript. To improve their detailed understanding of the frequently used reporting guidelines in nursing, and to increase general awareness about reporting guidelines, reviewers should acquire updated information. Opportunities in academic communities to actively share the experience of using reporting guidelines would be a practical measure to this aim, through events such as conferences and workshops.

Finally, the editorial board of journals should include the link to the EQUATOR Network website as well as frequently used reporting guidelines in the ‘Instructions for Authors’ to find the available reporting guidelines easily. In addition, the editorial board should attempt to provide clear instructions on the appropriate use of the guidelines so that authors and reviewers can easily assess the quality of the manuscript based on reporting guidelines. Journal editors and reviewers should also review the manuscript carefully, verifying the adherence to recognized reporting guidelines pertinent to the research design in the manuscript. Similar to the previous research conducted by the editorial board of KJWHN to evaluate reporting guideline use, further evaluation studies must be conducted regularly to identify reporting areas for improvement and weakness. These efforts should be actively shared with authors and reviewers to promote their understanding and motivation for using reporting guidelines. As noted above, continuous education for guideline use in the academic conference or workshop should be provided to authors and reviewers to improve research quality in the journals.

## Figures and Tables

**Table 1.** Summaries of the reporting guidelines commonly mentioned in the nursing journals (N=94)

Main study type	Reporting guideline acronym	Meaning of acronym	Applied studies or research design	Reporting guideline (year)	No. of journals mentioned	EQUATOR Network web address	Reporting guideline web address
Randomized trials	CONSORT	CONsolidated Standards Of Reporting Trials	Parallel group randomized trials	CONSORT 2010 (2010)	64	https://www.equator-network.org/reporting-guidelines/consort/	http://www.consort-statement.org/
				CONSORT 2001 (2001)			
Randomized trials (extensions)	TREND	Transparent Reporting of Evaluations with Nonrandomized Designs	Reporting of intervention evaluation studies using nonrandomized designs	TREND (2004)	27	https://www.equator-network.org/reporting-guidelines/improving-the-reporting-quality-of-nonrandomized-evaluations-of-behavioral-and-public-health-interventions-the-trend-statement/	https://www.cdc.gov/trendstatement/
							
Systematic review	PRISMA	Preferred Reporting Items for Systematic reviews and Meta-Analyses	Reporting systematic reviews and meta-analyses	PRISMA 2020 (2021)	71	https://www.equator-network.org/reporting-guidelines/prisma/	http://www.prisma-statement.org/
				PRISMA 2009 (2009)			
				QUOROM			
Systematic review (extensions)	MOOSE	Meta-analysis Of Observational Studies in Epidemiology	Meta-analysis of observational studies in epidemiology	MOOSE (2000)	12	https://www.equator-network.org/reporting-guidelines/meta-analysis-of-observational-studies-in-epidemiology-a-proposal-for-reporting-meta-analysis-of-observational-studies-in-epidemiology-moose-group/	-
Observational studies	STROBE	STrengthening the Reporting of OBservational studies in Epidemiology	Observational studies in epidemiology (cohort, case-control studies, cross-sectional studies)	STROBE (2007) STROBE (2005) STROBE (2004)	41	https://www.equator-network.org/reporting-guidelines/strobe/	https://www.strobe-statement.org/
Qualitative research	COREQ	COnsolidated criteria for REporting Qualitative research	Qualitative research for interviews and focus groups	COREQ (2007)	37	https://www.equator-network.org/reporting-guidelines/coreq/	-
Qualitative research	SRQR	Standards for Reporting Qualitative Research	Reporting of qualitative research studies	SRQR (2014)	14	https://www.equator-network.org/reporting-guidelines/srqr/	-
Diagnostic/	STARD	STAndards for the Reporting of Diagnostic accuracy studies	Studies of diagnostic accuracy	STARD 2015 (2015)	19	https://www.equator-network.org/reporting-guidelines/stard/	-
prognostic studies				STARD (2003)			
Case reports	CARE	CAse REports	For completeness, transparency and data analysis in case reports and data from the point of care	CARE (2013)	14	https://www.equator-network.org/reporting-guidelines/care/	http://www.care-statement.org/
Quality improvement studies	SQUIRE	Standards for QUality Improvement Reporting Excellence	Quality improvement in health care	SQUIRE 2.0 (2015)	42	https://www.equator-network.org/reporting-guidelines/squire/	http://www.squire-statement.org/
				SQUIRE 1.0 (2008)			

EQUATOR: Enhancing the QUAlity and Transparency Of health Research.

## Supplementary materials

Further details on supplementary materials are presented online (available at https://doi.org/10.4069/kjwhn.2022.06.08.1).

Supplementary material 1. Journals indexed in the nursing category of the Social Science Citation Index as of Jun 1, 2022 (N=122).
