# Supplementary material for: Strategies to improve the quality of reporting nursing research
Source: Korean J Women Health Nurs. 2022 Jun 21;28(2):77–82. doi: 10.4069/kjwhn.2022.06.08.1 (PMC9334175; doi:10.4069/kjwhn.2022.06.08.1)
Supplement: Supplementary material 1. — Journals indexed in the nursing category of the Social Science Citation Index as of Jun 1, 2022 (N=122). [file kjwhn-2022-06-08-1suppl1.pdf]

Supplementary material 1. Journals indexed in the nursing category of the Social Science Citation Index as of Jun 1, 2022 (N=122)

Journal rank	Journal name	JCR Abbreviation	ISSN	eISSN	2020 JIF	JIF Quartile
1	International Journal of Nursing Studies	INT J NURS STUD	0020-7489	1873-491X	5.837	Q1
2	European Journal of Cardiovascular Nursing	EUR J CARDIOVASC NUR	1474-5151	1873-1953	3.908	Q1
3	Journal of Family Nursing	J FAM NURS	1074-8407	1552-549X	3.818	Q1
4	Birth-Issues in Perinatal Care	BIRTH-ISS PERINAT C	0730-7659	1523-536X	3.689	Q1
5	International Journal of Mental Health Nursing	INT J MENT HEALTH NU	1445-8330	1447-0349	3.503	Q1
6	Nurse Education Today	NURS EDUC TODAY	0260-6917	1532-2793	3.442	Q1
7	Journal of Nursing Management	J NURS MANAGE	0966-0429	1365-2834	3.325	Q1
8	Nursing Outlook	NURS OUTLOOK	0029-6554	1528-3968	3.250	Q1
9	Journal of Advanced Nursing	J ADV NURS	0309-2402	1365-2648	3.187	Q1
10	Journal of Nursing Scholarship	J NURS SCHOLARSHIP	1527-6546	1547-5069	3.176	Q1
11	Women and Birth	WOMEN BIRTH	1871-5192	1878-1799	3.172	Q1
12	Intensive and Critical Care Nursing	INTENS CRIT CARE NUR	0964-3397	1532-4036	3.072	Q1
13	Journal of Clinical Nursing	J CLIN NURS	0962-1067	1365-2702	3.036	Q1
14	Journal of Psychiatric and Mental Health Nursing	J PSYCHIATR MENT HLT	1351-0126	1365-2850	2.952	Q1
15	Journal of Tissue Viability	J TISSUE VIABILITY	0965-206X	1876-4746	2.932	Q1
16	Worldviews on Evidence-Based Nursing	WORLDV EVID-BASED NU	1545-102X	1741-6787	2.931	Q1
17	Nursing Ethics	NURS ETHICS	0969-7330	1477-0989	2.874	Q1
18	International Nursing Review	INT NURS REV	0020-8132	1466-7657	2.871	Q1
19	Journal of School Nursing	J SCH NURS	1059-8405	1546-8364	2.835	Q1
20	Australian Critical Care	AUST CRIT CARE	1036-7314	1878-1721	2.737	Q1
21	Cancer Nursing	CANCER NURS	0162-220X	1538-9804	2.592	Q1
22	Collegian	COLLEGIAN	1322-7696	1876-7575	2.573	Q1
23	European Journal of Cancer Care	EUR J CANCER CARE	0961-5423	1365-2354	2.520	Q1
24	Asia-Pacific Journal of Oncology Nursing	ASIA-PAC J ONCOL NUR	2347-5625	2349-6673	2.509	Q1
25	European Journal of Oncology Nursing	EUR J ONCOL NURS	1462-3889	1532-2122	2.398	Q1
26	Nursing Inquiry	NURS INQ	1320-7881	1440-1800	2.393	Q1
27	Clinical Simulation in Nursing	CLIN SIMUL NURS	1876-1399	1876-1402	2.391	Q1
28	Journal of Midwifery & Womens Health	J MIDWIFERY WOM HEAL	1526-9523	1542-2011	2.388	Q1
29	Journal of the American Psychiatric Nurses Association	J AM PSYCHIAT NURSES	1078-3903	1532-5725	2.385	Q1
30	Nursing Research	NURS RES	0029-6562	1538-9847	2.381	Q1
31	Midwifery	MIDWIFERY	0266-6138	1532-3099	2.372	Q2
32	Geriatric Nursing	GERIATR NURS	0197-4572	1528-3984	2.361	Q2
33	Advances in Skin & Wound Care	ADV SKIN WOUND CARE	1527-7941	1538-8654	2.347	Q2
34	Scandinavian Journal of Caring Sciences	SCAND J CARING SCI	0283-9318	1471-6712	2.340	Q2
35	Nursing in Critical Care	NURS CRIT CARE	1362-1017	1478-5153	2.325	Q2
36	Seminars in Oncology Nursing	SEMIN ONCOL NURS	0749-2081	1878-3449	2.315	Q2
37	BMC Nursing	BMC NURS	1472-6955	1472-6955	2.283	Q2
38	Nurse Education in Practice	NURSE EDUC PRACT	1471-5953	1873-5223	2.281	Q2
39	Applied Nursing Research	APPL NURS RES	0897-1897	1532-8201	2.257	Q2
40	Research in Nursing & Health	RES NURS HEALTH	0160-6891	1098-240X	2.228	Q2
41	American Journal of Nursing	AM J NURS	0002-936X	1538-7488	2.220	Q2
42	Archives of Psychiatric Nursing	ARCH PSYCHIAT NURS	0883-9417	1532-8228	2.218	Q2
43	Perspectives in Psychiatric Care	PERSPECT PSYCHIATR C	0031-5990	1744-6163	2.186	Q2
44	Oncology Nursing Forum	ONCOL NURS FORUM	0190-535X	1538-0688	2.172	Q2
45	Journal of Pediatric Nursing-Nursing Care of Children & Families	J PEDIATR NURS	0882-5963	N/A	2.145	Q2
46	International Emergency Nursing	INT EMERG NURS	1755-599X	1878-013X	2.142	Q2
47	International Journal of Older People Nursing	INT J OLDER PEOPLE N	1748-3735	1748-3743	2.115	Q2
48	Journal of Professional Nursing	J PROF NURS	8755-7223	1532-8481	2.104	Q2
49	Asian Nursing Research	ASIAN NURS RES	1976-1317	2093-7482	2.085	Q2
50	Journal of Cardiovascular Nursing	J CARDIOVASC NURS	0889-4655	1550-5049	2.083	Q2

(Continued to the next page)

Supplementary material 1. Continued

Journal rank	Journal name	JCR Abbreviation	ISSN	eISSN	2020 JIF	JIF Quartile
51	Nurse Educator	NURS EDUC	0363-3624	1538-9855	2.082	Q2
52	Clinical Nursing Research	CLIN NURS RES	1054-7738	1552-3799	2.075	Q2
53	International Journal of Nursing Practice	INT J NURS PRACT	1322-7114	1440-172X	2.066	Q2
54	CIN-Computers Informatics Nursing	CIN-COMPUT INFORM NU	1538-2931	1538-9774	1.985	Q2
55	Journal of Child Health Care	J CHILD HEALTH CARE	1367-4935	1741-2889	1.979	Q2
56	Advances in Neonatal Care	ADV NEONAT CARE	1536-0903	1536-0911	1.968	Q2
57	Western Journal of Nursing Research	WESTERN J NURS RES	0193-9459	1552-8456	1.967	Q2
58	Journal of Transcultural Nursing	J TRANSCULT NURS	1043-6596	1552-7832	1.959	Q2
59	International Journal of Qualitative Studies on Health and Well-Being	INT J QUAL STUD HEAL	1748-2623	1748-2631	1.947	Q2
60	Pain Management Nursing	PAIN MANAG NURS	1524-9042	1532-8635	1.929	Q2
61	Journal of Hospice & Palliative Nursing	J HOSP PALLIAT NURS	1522-2179	1539-0705	1.918	Q2
62	Nursing & Health Sciences	NURS HEALTH SCI	1441-0745	1442-2018	1.857	Q3
63	Journal of Emergency Nursing	J EMERG NURS	0099-1767	1527-2966	1.836	Q3
64	Issues in Mental Health Nursing	ISSUES MENT HEALTH N	0161-2840	1096-4673	1.835	Q3
65	Advances in Nursing Science	ADV NURS SCI	0161-9268	1550-5014	1.824	Q3
66	Journal of Pediatric Health Care	J PEDIATR HEALTH CAR	0891-5245	1532-656X	1.812	Q3
67	Contemporary Nurse	CONTEMP NURSE	1037-6178	1839-3535	1.787	Q3
68	Nursing Open	NURS OPEN	2054-1058	2054-1058	1.762	Q3
69	Journal of Wound Ostomy and Continence Nursing	J WOUND OSTOMY CONT	1071-5754	1528-3976	1.741	Q3
70	Journal of Nursing Administration	J NURS ADMIN	0002-0443	1539-0721	1.737	Q3
71	Journal of Nursing Education	J NURS EDUC	0148-4834	1938-2421	1.726	Q3
72	JOGNN-Journal of Obstetric Gynecologic and Neonatal Nursing	JOGNN-J OBST GYN NEO	0884-2175	1552-6909	1.716	Q3
73	Critical Care Nurse	CRIT CARE NURSE	0279-5442	1940-8250	1.708	Q3
74	Journal of Nursing Research	J NURS RES	1682-3141	1948-965X	1.682	Q3
75	Australian Journal of Rural Health	AUST J RURAL HEALTH	1038-5282	1440-1584	1.662	Q3
76	Journal of Perinatal & Neonatal Nursing	J PERINAT NEONAT NUR	0893-2190	1550-5073	1.638	Q3
77	Journal of Pediatric Oncology Nursing	J PEDIATR ONCOL NURS	1043-4542	1532-8457	1.636	Q3
78	Rehabilitation Nursing	REHABIL NURS	0278-4807	2048-7940	1.625	Q3
79	Journal of Nursing Care Quality	J NURS CARE QUAL	1057-3631	1550-5065	1.597	Q3
80	Research in Gerontological Nursing	RES GERONTOL NURS	1940-4921	1938-2464	1.571	Q3
81	Journal of Addictions Nursing	J ADDICT NURS	1088-4602	1548-7148	1.476	Q3
82	Public Health Nursing	PUBLIC HEALTH NURS	0737-1209	1525-1446	1.462	Q3
83	Revista Latino-Americana de Enfermagem	REV LAT-AM ENFERM	1518-8345	1518-8345	1.442	Q3
84	Japan Journal of Nursing Science	JPN J NURS SCI	1742-7932	1742-7924	1.418	Q3
85	Workplace Health & Safety	WORKPLACE HEALTH SAF	2165-0799	2165-0969	1.413	Q3
86	MCN-the American Journal of Maternal-Child Nursing	MCN-AM J MATERN-CHIL	0361-929X	1539-0683	1.412	Q3
87	Journal of the American Association of Nurse Practitioners	J AM ASSOC NURSE PRA	2327-6886	2327-6924	1.370	Q3
88	JANAC-Journal of The Association of Nurses in Aids Care	J ASSOC NURSE AIDS C	1055-3290	1552-6917	1.354	Q3
89	Critical Care Nursing Clinics of North America	CRIT CARE NURS CLIN	0899-5885	1558-3481	1.326	Q3
90	Journal of Renal Care	J RENAL CARE	1755-6678	1755-6686	1.294	Q3
91	Australasian Emergency Care	AUSTRALAS EMERG CARE	N/A	2588-994X	1.283	Q3
92	Nursing Philosophy	NURS PHILOS	1466-7681	1466-769X	1.279	Q4
93	Journal for Specialists in Pediatric Nursing	J SPEC PEDIATR NURS	1539-0136	1744-6155	1.260	Q4
94	Journal of Gerontological Nursing	J GERONTOL NURS	0098-9134	1938-243X	1.254	Q4
95	Journal of Neuroscience Nursing	J NEUROSCI NURS	0888-0395	1945-2810	1.230	Q4
96	Journal of Continuing Education in Nursing	J CONTIN EDUC NURS	0022-0124	1938-2472	1.224	Q4
97	International Journal of Nursing Knowledge	INT J NURS KNOWL	2047-3087	2047-3095	1.222	Q4
98	Nursing Clinics of North America	NURS CLIN N AM	0029-6465	1558-1357	1.208	Q4
99	Journal of Forensic Nursing	J FORENSIC NURS	1556-3693	1939-3938	1.175	Q4
100	Journal of Psychosocial Nursing and Mental Health Services	J PSYCHOSOC NURS MEN	0279-3695	1938-2413	1.098	Q4

(Continued to the next page)

https://doi.org/10.4069/kjwhn.2022.06.08.1

Supplementary material 1. Continued

Journal rank	Journal name	JCR Abbreviation	ISSN	eISSN	2020 JIF	JIF Quartile
101	Revista da Escola de Enfermagem da USP	REV ESC ENFERM USP	0080-6234	1980-220X	1.086	Q4
102	Nursing Economics	NURS ECON	0746-1739	N/A	1.085	Q4
103	Journal of Perianesthesia Nursing	J PERIANESTH NURS	1089-9472	1532-8473	1.084	Q4
104	Clinical Nurse Specialist	CLIN NURSE SPEC	0887-6274	1538-9782	1.067	Q4
105	Clinical Journal of Oncology Nursing	CLIN J ONCOL NURS	1092-1095	1538-067X	1.027	Q4
106	Journal of Trauma Nursing	J TRAUMA NURS	1078-7496	1932-3883	1.010	Q4
107	Holistic Nursing Practice	HOLIST NURS PRACT	0887-9311	1550-5138	1.000	Q4
108	Journal of Korean Academy of Nursing	J KOREAN ACAD NURS	2005-3673	2093-758X	0.984	Q4
109	Gastroenterology Nursing	GASTROENTEROL NURS	1042-895X	1538-9766	0.978	Q4
110	Journal of Community Health Nursing	J COMMUN HEALTH NURS	0737-0016	1532-7655	0.974	Q4
111	Nephrology Nursing Journal	NEPHROL NURS J	1526-744X	2163-5390	0.959	Q4
112	Orthopaedic Nursing	ORTHOP NURS	0744-6020	1542-538X	0.913	Q4
113	Nursing Science Quarterly	NURS SCI QUART	0894-3184	1552-7409	0.883	Q4
114	Assistenza Infermieristica E Ricerca	ASSIST INFERM RIC	1592-5986	2038-1778	0.804	Q4
115	JNP-Journal for Nurse Practitioners	JNP-J NURSE PRACT	1555-4155	1878-058X	0.767	Q4
116	Wound Management & Prevention	WOUND MANAG PREV	2640-5237	2640-5245	0.698	Q4
117	Research and Theory for Nursing Practice	RES THEOR NURS PRACT	1541-6577	1945-7286	0.688	Q4
118	AORN Journal	AORN J	0001-2092	1878-0369	0.676	Q4
119	Acta Paulista de Enfermagem	ACTA PAUL ENFERM	0103-2100	1982-0194	0.667	Q4
120	Pflege	PFLEGE	1012-5302	1664-283X	0.655	Q4
121	Australian Journal of Advanced Nursing	AUST J ADV NURS	0813-0531	1447-4328	0.647	Q4
122	Bariatric Surgical Practice and Patient Care	BARIATR SURG PRACT P	2168-023X	2168-0248	0.607	Q4

Web of Science. 122 journals in Nursing category of Social Science Citation Index in the Journal Citation Reports [Internet]. Clarivate: Web of Science Group; 2021 [cited 2022 June 1]. Available from: https://jcr.clarivate.com/jcr/browse-journals
Copyright (c) 2022 Clarivate
